# Bee Venom Phospholipase A_2_: Yesterday’s Enemy Becomes Today’s Friend

**DOI:** 10.3390/toxins8020048

**Published:** 2016-02-22

**Authors:** Gihyun Lee, Hyunsu Bae

**Affiliations:** Department of Physiology, College of Korean Medicine, Kyung Hee University, 1 Hoeki-Dong, Dongdaemoon-gu, Seoul 130-701, Korea; glee@khu.ac.kr

**Keywords:** bee venom, phospholipase A_2_, immunity

## Abstract

Bee venom therapy has been used to treat immune-related diseases such as arthritis for a long time. Recently, it has revealed that group III secretory phospholipase A_2_ from bee venom (bee venom group III sPLA_2_) has *in vitro* and *in vivo* immunomodulatory effects. A growing number of reports have demonstrated the therapeutic effects of bee venom group III sPLA_2_. Notably, new experimental data have shown protective immune responses of bee venom group III sPLA_2_ against a wide range of diseases including asthma, Parkinson’s disease, and drug-induced organ inflammation. It is critical to evaluate the beneficial and adverse effects of bee venom group III sPLA_2_ because this enzyme is known to be the major allergen of bee venom that can cause anaphylactic shock. For many decades, efforts have been made to avoid its adverse effects. At high concentrations, exposure to bee venom group III sPLA_2_ can result in damage to cellular membranes and necrotic cell death. In this review, we summarized the current knowledge about the therapeutic effects of bee venom group III sPLA_2_ on several immunological diseases and described the detailed mechanisms of bee venom group III sPLA_2_ in regulating various immune responses and physiopathological changes.

## 1. Introduction

Bee venom is a weapon that bees use to protect themselves. In humans, however, it has been used as an anti-inflammatory medicine to relieve pain and treat chronic inflammatory diseases such as rheumatoid arthritis and multiple sclerosis [1,2,3,4,5,6,7]. While bee venom itself causes nociceptive and neurotoxic effects, several recent studies have demonstrated that bee venom has radioprotective [8], antimutagenic [9], anti-inflammatory [7,10,11,12,13,14], anti-nociceptive [15,16,17] and anti-cancer [18,19,20,21,22,23,24] activities.

Bee venom represents a complex composition of polypeptides, enzymes, amines, lipids and amino acids [25,26,27]. Some of them have been shown to have anti-inflammatory and anti-nociceptive effects or toxic and detrimental effects, while some of them have both beneficial and adverse effects under different conditions. However, the functions of each component remain unclear. Group III secretory phospholipase A_2_ from bee venom (bee venom group III sPLA_2_) makes up 10%–12% of dry bee venom and is known to be the major allergen in bee venom [28]. As other types and groups of PLA_2_s have been detected in inflammatory sites, most studies have focused on the harmful effects of bee venom group III sPLA_2_ and attempted to find a way to block the harmful effects of bee venom group III sPLA_2_. Comparatively beneficial roles of bee venom group III sPLA_2_ have been less well studied.

Many PLA_2_s cause harmful effects, such as inflammation in humans, but there is also proof that some PLA_2_s, such as mouse group IID and mouse group V sPLA_2_s, have anti-inflammatory effects [29,30]. Indeed, there are more than 30 known PLA_2_s and each of them has its own characteristics and functions [31]. Herein we highlight the newest findings on the beneficial roles of bee venom group III sPLA_2_.

## 2. Bee Venom Group III sPLA_2_ as an Enzyme

PLA_2_ from bee venom belongs to the group III sPLA_2_ enzymes, and simultaneously can act as a ligand for specific receptors. To elucidate the exact role of bee venom group III sPLA_2_, both activities should be considered.

PLA_2_ catalyzes the hydrolysis of the sn-2 ester linkage of glycerophospholipids to liberate free fatty acids and lysophospholipids. The cellular functions of PLA_2_ include a modulation of the release of arachidonic acid and a generation of eicosanoids, which are potent inflammatory mediators [31]. Moreover, PLA_2_ plays a central role in host defense, differentiation, and membrane remodeling [31,32]. PLA_2_ enzymes can be found in a variety of organisms, such as bacteria [33], fungi [34,35], plants [36], insects [37,38,39], reptiles [40], and mammals [32]. The PLA_2_ superfamily can be subdivided into 16 groups based on structure homology, source, and localization [31]. The distribution of these groups includes sPLA_2_ (groups I, II, III, V, IX, X, XI, XII, XIII, and XIV), Ca^2+^-dependent cytosolic PLA_2_ (group IV), Ca^2+^-independent cytosolic PLA_2_ (group VI), platelet-activating factor acetyl hydrolases (group VII and VIII), lysosomal (group XV) and adipose-specific PLA_2_ group (XVI) [41].

The sPLA_2_ family represents a group of extracellular enzymes that are structurally homologous and Ca^2+^-dependent. To date, a set of approximately 11 sPLA_2_ genes have been identified in various animal species based on genomic searches for sPLA_2_ sequences [42]. Individual sPLA_2_ exhibits partially overlapping but unique tissue and cellular distributions and substrate selectivity, suggesting its distinct biological roles in activating different target substrates [31]. For example, group IB sPLA_2_s found in pancreatic juice are involved in lipid degradation of food, whereas group IIA sPLA_2_s are involved in antibacterial defense [31].

Mammalian group III sPLA_2_ is an atypical sPLA_2_ member. It is more homologous to bee venom group III sPLA_2_ than other mammalian sPLA_2_s [43]. Recent studies have suggested that mammalian group III sPLA_2_ can mediate atherosclerosis [44] and differentiation of sperm maturation [45]. In addition, this enzyme can regulate neuronal outgrowth and survival by activating AKT [46]. One report has suggested its potential utility as a pharmacological agent for Alzheimer’s disease via enhancing α-secretase-dependent amyloid precursor protein processing to regulate membrane fluidity [47]. The relationship between mammalian group III sPLA_2_ and cancer has also been suggested [48,49]. The functions of bee venom group III sPLA_2_ should be studied differently from the mammalian group III sPLA_2_. Mammalian group III sPLA_2_ and bee venom group III sPLA_2_ have structural similarity in the central domain. However, they are different in the *N*-terminal and *C*-terminal domain extensions. Mammalian group III sPLA_2_ is a multi-domain protein with a molecular weight of 55 kDa, whereas bee venom group III sPLA_2_ has a molecular weight of about 15–16 kDa. Mammalian group III sPLA_2_ has a 130-amino-acid *N*-terminal domain extension and a 219-amino-acid *C*-terminal domain extension which are functionally unknown [41]. Structurally they are not homologous to bee venom group III sPLA_2_.

## 3. Bee Venom Group III sPLA_2_ as a Ligand

In addition to their catalytic activities, certain types of sPLA_2_s have been shown to be able to bind to specific membrane receptors and act as ligands to elicit cellular signals independent of their enzymatic activities. It has been proposed that various *in vitro* biological responses to mammalian pancreatic group IB sPLA_2_ can be mediated via a specific binding site for cell proliferation [50] and vascular smooth muscle contraction [51]. Two main types (M-type and N-type) of sPLA_2_ receptors have been identified. The M-type sPLA_2_ receptors were first identified in skeletal muscle cells [52] while the N-type sPLA_2_ receptors were first identified in rat brain membranes [53]. *In vivo*, mice lacking the M-type receptor exhibited tolerance to endotoxic shock, suggesting a role of group IB sPLA_2_ in the progression of acute inflammatory response [54]. In addition to mammalian sPLA_2_, some venomous neurotoxic sPLA_2_s exhibit high affinity binding to unidentified membrane-binding sites on a putative N-type receptor with the possible function of directing neurotoxic sPLA_2_s to specific cellular targets, thereby playing a crucial mechanistic role in their bioactivities [55].

Several reports have emphasized the receptor-binding of bee venom group III sPLA_2_ for its functions. Nicolas *et al.* have reported that binding to a N-type receptor of bee venom group III sPLA_2_ is closely correlated with its neurotoxicity [56]. They have demonstrated that mutants of bee venom group III sPLA_2_ with low affinity for N-type receptors are devoid of neurotoxic properties, even though some of them have retained high enzymatic activity. Meanwhile, Palm *et al.* have demonstrated that IL-33 receptor ST2 knockout mice have lower T helper type 2 (Th2) responses to bee venom group III sPLA2 compared to wild-type mice, suggesting that ST2 can mediate Th2 responses induced by bee venom group III sPLA2, although direct binding of bee venom group III sPLA2 onto ST2 has not been proven [57]. They have demonstrated that Th2 responses induced by bee venom group III sPLA_2_ are largely dependent on MyD88 expression in T cells. They have ruled out the contribution of TLRs and cytokines such as IL-1 and IL-18 as critical for the initiation and propagation of Th1 and Th17 responses. Additionally, bee venom group III sPLA_2_ has been reported to be a ligand for mannose receptor CD206 [58]. In our experiments, CD206 was found to be required for the immunomodulatory effects of bee venom group III sPLA_2_ (Equilibrium dissociation constant: 4.79 × 10^−6^ M) [59,60]. (A detailed explanation can be found in Section 5.1.).

## 4. Bee Venom Group III sPLA_2_: Yesterday’s Enemy

### 4.1. T Cell Responses and Anaphylaxis Induced by Bee Venom Group III sPLA_2_

Venoms from various species can induce Th2 and IgE responses and therefore represent a major class of allergens [25,61,62]. Type 2 responses to bee venom have been well documented in both mice and humans [25,61,63]. Recently, group III sPLA_2_ has been known as an “anaphylactic sPLA_2_” that can promote mast cell maturation and, consequently, anaphylaxis. This enzyme is a long-sought sPLA_2_ that can contribute to the regulation of mast cells and elicit mast cell activation in mouse skin [64], similarly to bee venom group III sPLA_2_. Transgenic overexpression of human group III sPLA_2_ led to spontaneous skin inflammation [65]. Dudler *et al.* have shown that bee venom group III sPLA_2_, but not its catalytically inactive variants, is able to induce the release of IgE-independent mediators including IL-4 from rodent mast cells [66]. When injected into mouse skin, bee venom group III sPLA_2_ can induce Th2 cell-type responses and group 2 innate lymphoid cell activation via enzymatic cleavage of membrane phospholipids and the release of IL-33 [57]. Mustafa *et al.* have reported that human basophils cannot release histamine in response to bee venom group III sPLA_2_; however, these human basophils can produce leukotrienes that may play an important role in the anaphylactic response [67]. Recently, Bourgeois *et al.* have shown that bee venom group III sPLA_2_ can induce CD1a-restricted T cell responses by releasing free fatty acids, resulting in IFN-γ production *ex vivo* [68].

### 4.2. Nociceptive Effects and Neurotoxicity of Bee Venom Group III sPLA_2_

It has been previously reported that PLA_2_s can affect a range of cells related to nociception [69]. sPLA_2_s are involved in pronociceptive glutaminergic neurotransmission in the substantia gelatinosa of the dorsal horn of the spinal cord. However, bee venom group III sPLA_2_ itself has no such effect on it at 0.1 unit/mL [70]. It has been reported that bee venom group III sPLA_2_ has nociceptive effects. For example, subplantar injection of bee venom group III sPLA_2_ can induce hind paw edema which was characterized by rapid onset and short duration (within 180 min) [71].

sPLA_2_ of doubtful origin can contribute to delayed *in vitro* and *in vivo* neurotoxic effects [72]. Nicolas *et al.* have demonstrated that an N-type receptor related to neurotoxicity could be exerted by bee venom group III sPLA_2_ *in vivo* [56]. When bee venom group III sPLA_2_ was injected directly into the cervical dorsolateral funiculus of a rat, it caused dose-dependent demyelination, and loss of oligodendrocytes, astrocytes, and axonopathy [73,74]. Titsworth *et al.* have suggested that blocking the activity and expression of group III sPLA_2_ may represent a novel and more efficient way to block multiple damaging pathways, thereby achieving better tissue protection and functional recovery [74].

## 5. Bee Venom Group III sPLA_2_: Today’s Friend

### 5.1. Anti-Inflammatory Effects of Bee Venom Group III sPLA_2_

**Figure 1:**
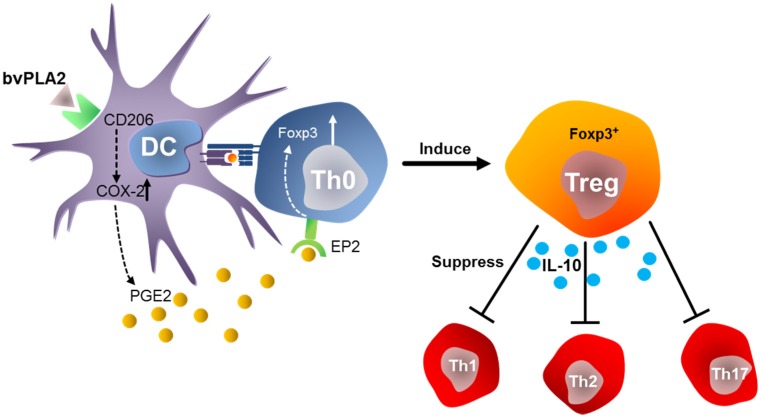
A model for the mechanism of action of bee venom group III sPLA_2_ in Foxp3^+^ Treg differentiation. Bee venom group III sPLA_2_ binds to CD206 of dendritic cells. CD206 signaling upregulates COX-2 expression, results in increased PGE2 secretion of dendritic cells. PGE2-EP2 signaling promotes immune regulation through Treg differentiation. bvPLA_2_: bee venom group III sPLA_2_.

Our lab has had a long-standing interest in studying the effects of bee venom on immunity. We have found that bee venom and its active compound bee venom group III sPLA_2_ can promote CD4^+^CD25^+^ regulatory T (Treg) cell differentiation [60]. A robust body of evidence has indicated that Treg cells can suppress the development of autoimmune diseases, such as rheumatoid arthritis and multiple sclerosis [75]. In addition to playing a role in autoimmune diseases, Treg cells have regulatory functions in transplantation tolerance, tumor immunity, allergy, and infection [76]. We have demonstrated Treg cell–mediated anti-inflammatory effects of bee venom and bee venom group III sPLA_2_ in diverse animal models of human diseases as well as its mechanism of action (Figure 1). Bee venom group III sPLA_2_ can directly bind to CD206 on the surface of dendritic cells and consequently promote the secretion of prostaglandin E2, resulting in Treg cell differentiation via EP2 receptor signaling in CD4^+^ T cells [60]. Bee venom group III sPLA_2_ can exert protective effects on airway inflammation via Treg cells in the mouse model of asthma [77]. Anti-inflammatory effects of bee venom group III sPLA_2_ have been demonstrated in cisplatin-induced renal injury and acetaminophen-induced liver injury [59,78]. These protective effects of bee venom group III sPLA_2_ against drug-induced organ toxicity are mediated by IL-10 production and Treg cell modulation. These findings are in agreement with previous observations that bee venom immunotherapy can increase Treg cell population and have a protective effect against allergy [79,80], although the relevance of bee venom group III sPLA_2_ in this response is unclear.

Palm *et al.* have demonstrated that bee venom group III sPLA_2_ injection conferred protective immunity [57]. They have demonstrated that IgE responses to bee venom group III sPLA_2_ can protect mice from future challenges with a near-lethal dose of bee venom group III sPLA_2_. Similarly, Marichal *et al.* have showed evidence that IgE-dependent immune responses against bee venom can enhance the survival of mice, supporting the hypothesis that IgE as a contributor to allergic disorders has an important function in host protection against noxious substances [81].

### 5.2. Anti-Neuronal Injury and Anti-Nociceptive Effects of Bee Venom Group III sPLA_2_

Current evidence suggests that mammalian group III sPLA_2_ may affect some neuronal functions, such as neuritogenesis, neurotransmitter release and neuronal survival [46,82]. Bee venom group III sPLA_2_ can prevent neuronal cell death and inflammation. Bee venom group III sPLA_2_ can inhibit prion protein fragment_106–126_-induced neuronal cell death [83]. Prion protein fragment_106–126_-mediated increase of p-p38 MAPK and cleaved caspases and decrease of p-AKT could be blocked by bee venom group III sPLA_2_ treatment. Recently, we have demonstrated that bee venom group III sPLA_2_ can promote the survival of dopaminergic neurons in a 1-methyl-4-phenyl-1,2,3,6-tetrahydropyridine mouse model of Parkinson’s disease. The neuroprotective effects of bee venom group III sPLA_2_ are associated with microglial deactivation and reduced CD4^+^ T cell infiltration [60].

Bee venom group III sPLA_2_ treatment also can strongly alleviate oxaliplatin-induced acute cold and mechanical allodynia in mice by activating the noradrenergic system via α2-adrenegic receptors, but not the serotonergic system [84].

### 5.3. Anti-Tumor Effects of Bee Venom Group III sPLA_2_

It has been reported that bee venom group III sPLA_2_ and phosphatidylinositol-(3,4)-bisphosphate can synergistically disrupt membrane integrity and cause subsequent cell death in renal cancer cells [85]. Putz *et al.* have reported that bee venom group III sPLA_2_ and phosphatidylinositol-(3,4)-bisphosphate can synergistically generate tumor lysates, thus enhancing the maturation of immunostimulatory human monocyte-derived dendritic cells [86]. Such tumor lysates represent complex mixtures of tumor antigens that can simultaneously exhibit potent adjuvant properties. In addition, they meet all requirements for a tumor vaccine. Increasing evidence has suggested a modulatory role for phospholipid dendritic cell differentiation, thereby affecting the immunogenic potential of antigen-presenting cells [87,88,89]. Phospholipids can exert their role either as precursors of second messengers or through direct action on intracellular signal transduction pathways [90,91]. It is possible that the anti-tumor effect of bee venom group III sPLA_2_ and phosphatidylinositol-(3,4)-bisphosphate may regulate cell survival, cell proliferation, and cell cycle [90,92,93]. Accordingly, Putz *et al.* have proposed that bee venom group III sPLA_2_ might also be responsible for bee venom-induced apoptosis [86]. However, there is a report showing that lysophospholipids can activate ovarian and breast cancers cells [94]. Ovarian cancer patients have increased levels of plasma lysophospholipids [95]. The effect of bee venom group III sPLA_2_ on cancer should be confirmed in each tissue.

### 5.4. Vaccination Approaches

Almunia *et al.* have used bee venom group III sPLA_2_ histidine-34 replacement with glutamine (bee venom group III sPLA_2_H34Q), which abolished its catalytic activity, to induce the cross-presentation of a peptide fused to it [96]. Bee venom group III sPLA_2_H34Q can allow continuous peptide loading by prolonged cross-presentation with as much antigen as is available in antigen-presenting cells [97]. In their experiment, it augmented at least eight-fold in the cross-presentation of peptide derived from the antigen and showed low immunogenicity, which are critical attributes for vaccine development. They suggested that bee venom group III sPLA_2_H34Q could be used as a membrane-binding vector to promote major histocompatibility complex (MHC) class I peptide cross-presentation and MHC class II peptide presentation for the preparation of cell-based vaccines.

### 5.5. Anti-Parasite and Anti-Bacterial Effects of Bee Venom Group III sPLA_2_

The power of bee venom group III sPLA_2_ as a host defense molecule can be observed by studying bacteria and parasites. It has been reported that bee venom group III sPLA_2_ has significant trypanocidal and antibacterial effects on Gram-negative bacteria [98]. Bee venom group III sPLA_2_ and snake venom sPLA_2_s can also significantly block the development of parasites in mosquito hosts [99]. Further study using transgenic mosquitoes expressing bee venom group III sPLA_2_ in the midgut has revealed a similar inhibitory effect on *Plasmodium* ookinetes [100].

## 6. Conclusions

Studies over the previous decades have advanced our knowledge about bee venom group III sPLA_2_. Herein, we summarized the adverse and beneficial effects of bee venom group III sPLA_2_ (Table 1) and discussed its underlying mechanisms. However, parts of the underlying mechanisms still remain unclear, although new experimental data have opened a window into harnessing the beneficial roles of bee venom group III sPLA_2_. We believe that bee venom group III sPLA_2_ can be used for therapeutic purposes if careful provisions are taken to avoid adverse effects.

The effect of bee venom group III sPLA_2_ is related in part to the tissue used and the experimental settings. The distribution of receptors or other targets including lipids is tissue-dependent. The sphere of activity due to bee venom group III sPLA_2_ must have been affected by the method of each experiment. For instance, the nociceptive effect by bee venom group III sPLA_2_ lasted for a short time (less than 180 min after bee venom group III sPLA_2_ injection) in the paw [71] whereas the anti-nociceptive effect by bee venom group III sPLA_2_ lasted for more than one day after bee venom group III sPLA_2_ intraperitoneal injection [84]. Neurotoxic and anti-neurotoxic effects of bee venom group III sPLA_2_ also could be related in part to experimental settings. It has been reported that bee venom group III sPLA_2_ can induce neuronal death when it is injected directly into the spinal cord [73,74]. However, it offered protection against neuronal death in the substantia nigra when it was injected into the peritoneal cavity [60].

To verify the necessity of enzymatic activity for the actions of bee venom group III sPLA_2_, most experiments have used heat or chemically inactivated bee venom group III sPLA_2_. However, it is possible that heat or chemical treatment for the inactivation of catalytic activity might have changed the protein structure of the bee venom group III sPLA_2_, resulting in altered effects on receptors. For example, manoalide-inactivated bee venom group III sPLA_2_ that has been widely used as a chemically inactivated bee venom group III sPLA_2_ lost its binding affinity on CD206 and enzymatic activity simultaneously (unpublished data from our lab). Using recombinant mutant bee venom group III sPLA_2_s, the Gelb group has demonstrated that receptor-binding and enzymatic activity are two independent molecular events [56,101]. To determine whether the effect of bee venom group III sPLA_2_ is mediated by its enzymatic activity or receptor-binding, experiments should be designed to compare its effect with mutants of bee venom group III sPLA_2_ that have receptor-binding activity without enzymatic activity. Additionally, the optimal dose and treatment method to be used without adverse effects should be determined in further experiments, including preclinical and clinical studies.

## Figures and Tables

**Table 1:** Adverse and beneficial effects of bee venom group III sPLA_2_.

**Adverse Effects**	**Specific Effects**	**Experimental System**	**Dose**	**Reference**
Induction of Type 2 responses	Promote Th2 differentiation and ILC2 activation	mouse, *in vivo*, s.c. injection or mouse, *in vivo*, i.p. injection for 3 consecutive days	50–100 µg/mouse	(Palm *et al.*, 2013) [57]
Nociceptive effects	Induce paw oedema for less 3 h	rat, *in vivo*, injection into paw	30 µg/paw	(Landucci *et al.*, 2000) [71]
Neurotoxicity	Induce neuronal death	rat, *in vivo*, microinjection into spinal cord	0.05–0.5 µg/rat	(Liu *et al.*, 2006) [73]
	Create demyelination and remyelination	rat, *in vivo*, microinjection into spinal cord	1.5–6 ng/rat	(Titsworth *et al.*, 2007) [74]
**Beneficial Effects**	**Specific Effects**	**Experimental System**	**Dose**	**Reference**
Anti-inflammatory effects	Promote Treg differentiation	mouse, *in vivo*, i.p. injection	0.1–1 mg/kg	(Chung *et al.*, 2015) [60]
	Supress airway inflammation	mouse, *in vivo*, i.p. injection	0.2 mg/kg	(Park *et al.*, 2015) [77]
	Protect cisplatin-induced renal inflammation	mouse, *in vivo*, i.p. injection	0.2 mg/kg	(Kim *et al.*, 2015) [59]
	Protect acetaminophen-induced liver inflammation	mouse, *in vivo*, i.p. injection	0.2 mg/kg	(Kim *et al.*, 2014) [78]
Anti-nociceptive effects	Reduce oxaliplatin-induced neuropathic pain	mouse, *in vivo*, i.p. injection	0.2 mg/kg	(Li *et al.*, 2015) [84]
Anti-neuronal injury	Prevent MPTP-induced neurotoxicity	mouse, *in vivo*, i.p. injection	0.2 mg/kg	(Chung *et al.*, 2015) [60]
	Inhibit PrP(106–126)-induced neuronal cell death	human neuroblastoma cell lines (SH-SY5Y), *in vitro*	50 nM	(Jeong *et al.*, 2011) [83]
Anti-tumor effects	Inhibit growth of various cancer cell lines synergistically with PtdIns(3,4)P2	A498, DU145, BEAS-2B, T-47D cell lines, *in vitro*	10 µg/mL	(Putz *et al.*, 2006) [86]
	Inhibit A498 cell line growth synergistically with PtdIns(3,4)P2	human kidney carcinoma cell line (A498), *in vitro*	10 µg/mL	(Putz *et al.*, 2006) [85]
Anti-parasite effects	Inhibit ookinete binding on mosquito midgut	mosquito, *ex vivo*	3.2 µM	(Zieler *et al.*, 2001) [99]
